# Challenges of emergency evacuation of residential areas caused by chemical release due to the earthquake: a Natech event scenario

**DOI:** 10.5249/jivr.v14i1.1698

**Published:** 2022-01

**Authors:** Parvin Shafiei Moghaddam, Katayoun Jahangiri, Sanaz Sohrabizadeh, Nemat Hassani, Mohammad Hoseini Moghaddam, Ghazaleh Monazami Tehrani

**Affiliations:** ^*a*^ Department of Health in Disasters and Emergencies, School of Public Health and Safety, Shahid Beheshti University of Medical Sciences, Tehran, Iran.; ^*b*^ Department of Rescue and Relief and Disaster Management, Iran Helal Institute of Applied Science and Technology, Tehran, Iran.; ^*c*^ Air Quality and Climate Change Research Center, Shahid Beheshti University of Medical Sciences, Tehran, Iran.; ^*d*^ Department of Civil, Water & Environmental Eng., Shahid Beheshti University, Tehran, Iran.; ^*e*^ Department of Prospective Studies, Institute for Social and Cultural Studies (ISCS), Tehran, Iran.; ^*f*^ Department of Health, Safety and Environment, School of Public Health and Safety, Shahid Beheshti University of Medical Sciences, Tehran, Iran.

**Keywords:** Emergency Evacuation, Earthquake, Chemical Release, Community Health, Natech

## Abstract

**Background:**

In recent decades, earthquakes, as natural hazards that caused direct effects both on communities and the chemical industry, produced many Natech events. Natech term is utilizing to describe the technological disasters caused by natural hazards. This study was conducted on the emergency evacuation challenges of residential areas adjacent to a refinery near Tehran based on H_2_S toxic gas release following a possible earthquake scenario.

**Methods:**

This Research was an applied study at two phases in 2020. In the first phase, a review study was conducted to identify the community's previous experiences on emergency evacuation following Natech events. In the second phase, the challenges of emergency evacuation were analyzed based on the scenario of a possible earthquake and gas release from the refinery.

**Results:**

Due to the high seismic vulnerability of structures in the area affected Natech risk, the total Resident population in this area would be affected simultaneously by an earthquake and H_2_S gas release in concentration 30 ppm as the result of the earthquake impact on chemical facilities. Emergency evacuation would be inevitable. The existing evacuation places are very unsafe and dangerous due to having open spaces. The nearest suitable evacuation places were found in the north direction for more than 38 % of the exposed population and in the east, west, and south direction for more than 61% of them.

**Conclusions:**

The emergency evacuation challenges were discussed in 4 viewpoints, disrupted or interrupted rescue and firefighting operation, unnecessary evacuation, frequent evacuation, and evacuation behavior. The measures such as revising and updating emergency evacuation maps; public informing, training, preparedness; providing protocols and training for operational and therapeutic response teams; and coordination improvement can help resilience increasing to such disasters.

## Introduction

In recent decades, earthquakes as natural hazards had direct effects both on communities and the chemical industry, especially refineries and petrochemicals, as well caused many Natech events. The term Natech was used for the first time by Schwartz and Meyer to describe technological disasters caused by natural hazards,^1^ and later it became common among other researchers.^2-7^ Natech Events often are associated with fire, explosion, or toxic substance release.^8^ Natech events are complicated due to hazardous substances release and affecting emergency response capacities by natural hazards.^9^ Hence, Natech event management of the release of hazardous materials following an earthquake is complex. The Sendai framework has emphasized the importance of integrated Risk management of all hazards and challenges of such events; too.^10^ The emergency evacuation of the affected residential areas is the most important measure in these events. Valuable experiences and lessons have been provided about the evacuation challenges from Studying 102 Natech events caused by the release of hazardous materials that led to the evacuation of residential areas in the United States in the period 1990-1990,^5^ the Izmit earthquake in Turkey in 1999,^11-13^ the Great East Japan earthquake and tsunami in 2011.^3,8,14^

Due to the experience of previous destructive earthquakes, high population density, and proximity of residential areas to hazardous chemical industries in Tehran, the Natech events risk assessment following the possible earthquake and the toxic substances release, fire, and explosion should be considered in preparedness and emergency response plans. 

For this purpose, the present study investigated the emergency evacuation challenges of residential areas in the south of Tehran based on the Natech event scenario of H_2_S Toxic gas releases from a refinery following a possible earthquake and its impact on the urban community.

## Methods 

**Study Design**

This applied study has been conducted to investigate the challenges of emergency evacuation of the urban community due to the release of toxic gas from a refinery following the scenario of a possible earthquake in 2020.

**Setting**

Iran is located in the Alpine-Himalayan seismic belt.^15^ Tehran, the capital of Iran, is known as one of the 20 metropolises in the world, as well as one of the 17 metropolises with a population of over 10 million people that is among 3 active faults and in a region with a high relative risk for earthquakes.^16-18^ Among the mentioned faults, the North Tehran and North Rey faults are the most important active and inverse ones in the region.^17^ Examining the documents and seismic records of the faults of the south of Tehran revealed the occurrence of a high number of powerful and destructive earthquakes (magnitudes of 7.1, 7.2, and 7.6 on the Richter scale) in the shahre-Rey that has been associated with many casualties.^15-17,19^

The refinery under research is located in the south of Tehran, between the northern and southern Rey-Ivanki faults in the north and the Kahrizak-Pishva faults in the south. Tehran has 22 municipality districts. In recent decades, increasing population, urbanization, and the gradual development of residential areas in the north (District 20), northeast (District 20 suburb areas), and west (District 19 suburb areas) have caused the proximity of these areas to the refinery and other chemical industrial facilities.

**Study steps**

After a preliminary search and review of references of key papers, the Web of Science, PubMed, and Scopus databases were used to search and investigate emergency evacuation challenges in the community after the Natech events. The articles, guidelines, books, and conference papers had been published in either Persian or English from 1980 to 2020 and related to industrial disasters and especially chemical disasters after earthquakes. The reason for choosing the beginning of this period was the increasing trend of natural disasters and its various consequences on the communities and the proclamation of the 1980s as the decade of disaster risk reduction by the United Nations.^20^ Search terms and keywords were selected by taking the opinions of experts in the disaster management field, and then the search strategy was compiled as follows: 

[Natech AND ("Natural Hazard" OR Earthquake) AND ("Chemical Release" OR "Hazmat Release" OR "Toxic Release" OR "Industrial Release") AND ("Oil Refinery" OR "Petrochemical Industries" OR "Chemical Industries") AND ("Response Capacity" OR Search & Rescue OR Evacuation)]

**Data Collection Tools and Methods**

In this study, the "Worst-Case Scenario" in a dense plume of RMP (or the US EPA-1999 Risk Management plan Guide)^21^ was utilized to determine the "End-point distance" of H_2_S gas release at the ERPG-2 concentration. Rapid-N software supported this guide and to be used to simulate atmospheric gas dispersion. The last version of the National census statistic (2016) was used to extract information about the number of populations and households at-risk for emergency evacuation.^22^ As well, wherever it was required, we used the latest information on neighborhood population and households to be published by the municipality (2021), emergency evacuation maps.^23^ And previous studies were used to determine the features of urban districts.^16-18,24-26^

**Natech Event Scenario**

The scenario of the activation of the Ray fault in the south of Tehran with the magnitude: Mw= 7.5 (equivalent to MMI: 10 (X) in the modified Mercalli scale) was designated as the consequence analysis criterion for the release of H_2_S toxic gas into the adjacent area in off-site the facility following an earthquake. Every refinery produces a lot of harmful substances in its process, which is often a liquid state and has the potential to catch fire and explode. The main aim of the present study was the assessment of Natech risk consequences due to the release of toxic gases on the communities around the refinery. Hydrogen sulfide gas had selected by experts, because it is gaseous at atmospheric temperature, heavier than air, and its toxicity,^21,27^ quantity, and concentration were such that if released, it was considered a health hazard to surrounding communities.

The EPA guide described the "worst-case scenario" based on the assumptions including the total quantity of the gaseous released in 10 minutes, dense plume, and urban topography, as well as the meteorological conditions atmospheric stability class F (stable atmosphere) and wind speed of 1.5 meters per second (3.4 miles per hour), the ambient air temperature of 25 C (77 F), and Relative humidity of 50%^21^ In such conditions, the gas dispersed in all directions from the damaged vessel, which appears circularly on the map. Obviously, as the distance from the vessel increases, the gas concentration would decrease to the side the end-point distance. In a stable atmosphere situation, the exposure of people to gas would become longer and contains serious health effects. In addition, this scenario is consistent with the conditions of Tehran that are faced with frequent and continuous weather stability, especially in the southern parts of the city in some months of the year.

**Figure 1:**
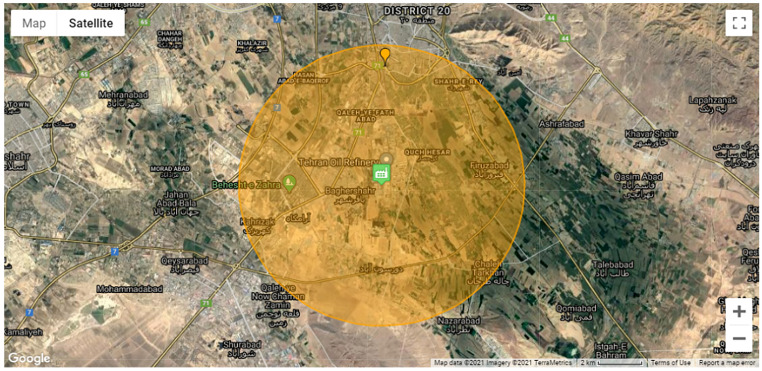
Natech risk map for H_2_S release in research area.

In this regard, Figure 1 shows the Natech risk map and the geographical extent of the area influenced by exposure to the released H_2_S gas at the ERPG-2 concentration with a radius of 6.5 km from the vessel. Natech risk assessment performed by N-Rapid software. The software analyzes the earthquake hazard and faults parameters, types of equipment failure curves, chemical substances physical and chemical properties. In addition, this software supports EPA, and in this Risk Management Plan Guide, toxic gas Endpoint is determined based on ERPG-2 concentration. The H_2_S data as toxic gas has shown in the following Table 1.

**Table 1:** Data for H_2_S as Toxic Gases.^21^

CAS Number	Chemical Number	Molecular Weight	Ratio of Specific Heats	Data for Toxic Gases Toxic Endpoint			Liquid Factor Boiling (LFB)	Density Factor (DF) (Boiling)	Gas Factor (GF)K	Vapor Pressure@ 25 C (psi)	Reference Table b
				mg/L	ppm	Basis					
6/4/7783	Hydrogen sulfide	34.08	1.32	0.042	30	ERPG-2	0.13	0.51	20	302	Dense

According to the AIHA (2007), the ERPG-2 value is the maximum concentration in the air below which nearly all individuals can be exposed for up to 1 hour without experiencing or developing irreversible or other serious health effects or symptoms impairing an individual's ability to take protective actions.^28^

Natech risk map was placed on Tehran’s map and the urban districts, suburb areas, population, and households affected were determined to emergency evacuation operation. Finally, emergency evacuation capacity was analyzed with emphasis on safe evacuation places and wide and safe evacuation routes in the Natech risk area.

**Ethical consideration**

This study was a part of a doctoral dissertation approved by Shahid Beheshti University of Medical Sciences, Tehran, Iran under the code of IR. SBMU. PHNS.REC.1398.040.

## Results

**Evacuation capacity**

The findings of this study have assessed the challenges of emergency evacuation based on the JICA-2004 recommendation^24^ with emphasis on the two parameters of safe evacuation places and safe and wide evacuation routes.

**Emergency evacuation**

Locating the Natech event risk map upon the geographical map of Tehran shows that the southern regions of the city, especially some parts of districts 19 and 20, are exposed to H_2_S gas release with the ERPG-2 concentration (i.e., 30 ppm) (Table 2). Evidence was shown that if the Rey faults are activated, the entire south of Tehran is exposed to considerable structural damage. The rate of damage to residential buildings in District 20, which is located between the two faults of the north and south of Rey, will reach 78.6% depending on the types of buildings and the type of materials used. On the other hand, population density and a large number of vulnerable and poorly structured (semi-skeleton from brick and iron) buildings, can lead to the destruction of about 350,000 residential units. This means that the entire those who are trapped under the rubble of damaged buildings and even those who live in the buildings rescued from the earthquake are at the risk of exposure to H_2_S toxic gas. 

**Table 2:** The number of residents (people and households), safe locations, and evacuation directions in separate urban areas affected by the Natech risk.^22,23^

District	Area	Neighborhood	Population	Family	Number of safe places	Evacuation direction	Forced to evacuate			
			
20	5	6	91937	24373	31		Population		Household	
							number	%	number	%
20	5	Estakhr	12509	4024	6	North	52035	38.4	16771	43
	5	Beheshti	9201	3164	3	North				
	5	Sar Takht	5699	1820	3	North				
	5	Hashem Abad	14245	4515	3	North				
	5	Vali Abad	10381	3248	6	North				
	5	Alaedin	2629	847	10	East	40477	29.8	12802	32.8
	6	Abbas Abad	17625	5985	6	East				
	6	Taqi Ababd	7308	2177	3	East				
	6	Suburb	12915	3793	-	East				
	7	Suburb	3322	800	-	South	3322	2.4	800	2
19	4	Suburb	25100	6,100	-	West	40094	29.6	8559	22
	5	Suburb	14994	2499	4	West				
Total			135478	38972	44					

Health Infrastructure such as other residential buildings would be vulnerable. Even in undamaged structures by the earthquake, the entire staff and patients of these centers must be evacuated due to permeable to the air contaminated with the toxic gas. 

Results found one-fourth of health infrastructure types and one-third of firefighting stations as disaster responder organizations were at-risk to earthquake and Natech events. In addition, half of the accommodation center and about one-third of the subway station, during the night and day, respectively, as crowd places were at-risk the Natech events. In this study, the subway stations were one of the most dangerous locations for Natech events because hydrogen sulfide is heavier than the air and remains on the ground and inhaled surfaces, and tends to penetrate indoor and enclosed spaces, especially at low-level (Table 3). In this regard, one-third of educational centers and more than 21.5% of the population under the age of 15 as a vulnerable segment of society with 29127 people will be at-risk to the Natech event. However, over 5.5% of the population above the age of 65 as other vulnerable segments of society with 7451 people would be at-risk to the toxic gas. This group often suffers from some diseases such as pulmonary and cardiovascular disorders and poor physical conditions. These problems act as barriers to rapid evacuation and solitary so that, in many cases, the elderly need help. The population and infrastructure of the affected area of the earthquake and Natech risk in the city districts have illustrated in Table 2.

**Table 3:** The population and infrastructure of the affected area by the earthquake and Natech risk in the city districts.

variable	Earthquake Risk		Natech Risk (6.5 km)	
			
**District **	20	19	20	19
**Area**	7	5	3	2
**Neighborhood**	20	15	8	0
**Hospital**	2	0	0	0
**Clinic**	35	21	17	1
**Health home**	21	15	8	1
**Medical & Health Centers**	18	11	3	1
**Health Centers**	3	7	1	2
**Educational Centers**	36	55	12	5
**Accommodation Centers**	7	1	4	0
**Subway Station**	7	10	1	5
**Firefighting Station**	6	6	2	2
**Disaster Management base**	10	5	5	0
**Emergency Evacuation Location**	120	95	40	4
**Population**	453740	287024	135478	40094
**Households**	135034	77764	38972	8599

Emergency evacuation to the neighboring areas will be inevitable for all exposed residents of districts 19 and 20. It will create new challenges for the host region, which had been severely affected by the earthquake and simultaneously had to respond to the needs of people from other areas. Managing emergency evacuation operations, a large number of people and households who are exposed to toxic gas release in the affected area is a challenging issue, especially in a short time.^29^ In this situation, the responsible institutions had provided the budget, plans, and execute strategies for providing the equipment necessary to respond to an earthquake and training experienced manpower to perform emergency response operations, including search and rescue, accommodation, emergency evacuation, and periodic practices to increase the skills of their operations teams. While, no budget, plans, and executive measures were considered for evacuation operations in Natech events.

Evacuation routes analysis were shown the obstruction of urban narrow passages in district 20 with the debris of buildings destroyed by the earthquake. The disruption, delay, and slowing in the transportation system would be the serious problems in the emergency evacuation operation process caused by the routes obstruction. It can lead increase in the exposure time to toxic gas in residents, those trapped under the debris, and response teams and lead to more casualties and health effects. Although there are suitable 44 safe evacuation locations for an earthquake in the area, they would be very unsafe and dangerous during the release of toxic gas due to open spaces.

The findings showed that the nearest evacuation suitable places to be located in the east direction for near 29% of the exposed population and the east, west, and south direction for more than 61% of them. While for more than 34% of the remained population, the nearest places were located in the north direction that the vulnerability risk of their buildings is very high. The geographical direction of the emergency evacuation due to Natech risk based on population and household has illustrated in Table 2.

## Discussion

**Evacuation Capacity Challenge**

Findings showed the entire exposed population to Natech events risk (released H_2_S gas) must evacuate. The emergency evacuation capacity was investigated based on the two determinants of safe evacuation places and safe and wide evacuation paths. The Research has found that all existing evacuation places and routes are unsafe and dangerous due to their open spaces and blockages paths. Emergency evacuation is a challenging issue in Natech events risk management.^29^ Despite, numerous studies that have been conducted on emergency evacuation in earthquakes, but few studies were focused on emergency evacuation in multi-hazard risks, especially Natech events risk. Studying Natech events shows that out of 102 evaluated disasters that led to evacuation, only 15% were related to earthquakes, hurricanes, tornadoes, and floods; 35% were due to release in fixed facilities (22% in refineries); 25% were related to the release of natural gases, and finally, 21% were due to the release of petroleum products.^5^ In this study the challenges of emergency evacuation have been discussed from four dimensions: disrupted and ceased rescue and firefighting operations, unnecessary evacuation, frequent evacuation, and evacuation behavior.^29^

**Disrupted or Ceased Rescue and Firefighting Operations**

The firefighting and search and rescue operations ceased due to issuing a forced evacuation order, Just a few hours after the occurrence of the Izmit earthquake(1999) and the releases of toxic gases of acrylonitrile, and ammonia from the industries of acrylic fiber production and fertilizer production, respectively.^11,13,30^ Extending fire in the oil refinery and intentionally releasing the ammonia into the air (within 48 hours) to prevent an explosion caused speed issuing evacuation orders.^11,13,30^ The cessation of operations forced the search and rescue teams and people to leave the area for two days while they tried to save the lives of those trapped under the collapsed building.^11,13,30^ On the other hand, the chances of life of trapped survivors under rubble considerably have reduced due to prolonged inhalation of the released toxic gases result of evacuation orders.^11,13,30^ It may remain unclear so forever how many people would have been saved if the search operation had not been canceled.^11^ In addition, fear and leaving the duty of many firefighters has happened after issuing evacuation orders. Some reasons included lack or low knowledge about the properties of the released hazardous materials and lack of trained and exercised forces to respond to releases of chemical materials following an earthquake at the refinery. Finally, it led to a severe reduction in the firefighting capacity and leaving the fire out of control.^12^

**Unnecessary Evacuation**

In the Izmit earthquake in Turkey (1999), the evacuation order had issued to an area approximately 100 times larger than the request of the acrylic fiber plant and the refinery to evacuate an area within a radius of 1.2 km (45 km^2^)^12^ and 5 km (78 km^2^), respectively.^11-13^ Even the cities more than 10 km away from the release sources evacuated with the population equivalent to tens of thousands of people.^13^ The unnecessary evacuation has happened because of inadequate communication through informal channels, limited awareness, misunderstanding, the chaos caused by the earthquake.^13^ For this reason, many people who were exposed to toxic gases for more than 20 hours to informed of the evacuation order by local security forces.^13^

**Frequent Evacuations**

In the Great East Japan Earthquake and Tsunami (2011), it seems various reasons to issue several evacuation orders. The study found that two-thirds of the studied population evacuated and one-third equivalent to 32% of them had not evacuated. More than 64% of the people were evacuated based on the first evacuation order was issued for the earthquake and tsunami.^8,14^ The second was related to Natech events and the possibility of an LPG tank farm's explosion, fire spreading, sulfur ignition, and the formation of a toxic cloud,^3^ that led to forced evacuation near to 31% of people who lived in an area within a 2 km radius around the refinery.^8,14^ Despite the reduction of natural hazards, the second evacuation order had partly issued due to the earthquake or tsunami and another part due to overcrowded shelters or a shortage of essential items for the families whose members required special needs. And it included 21% and 9% of the evacuated, respectively.^8,14^

**Evacuation Behavior**

In confronted with the Natech risk, the evacuation behavior of households had studied via several variables such as risk perception, location, time of evacuation order announcement, warning source, demographic variables (age and household size), wind direction, and training. 

In the field of Natech risk management, the development of protection strategies largely is depended on the understanding of the evacuation behavior of households faced with such events by emergency managers.^14^ A study on the Greater East Japan Earthquake and Tsunami in 2011 found risk perception variables were a key factor in understanding the evacuation decision-making process.^8^ The size of the household variable was significantly related to the time of mobilization to evacuate so that the large households had more willing to quicker evacuation than small households, and it showed receiving an evacuation order can be reduced the household's response time to Natech threats.^8,14^ Wind direction was an influential factor in the risk understanding and the evacuation response of households to a Natech event.^8^ The Great East Japan Earthquake and Tsunami in 2011 revealed the lack of experience evacuating and training for the Natech events among the majority of residents.^8^

The age variable also was significantly related to the time of mobilization to evacuate,^8,14^ so that the evacuation probability was 1.22 times higher for each year of aging in Japan.^14^ The older people were more willing and quicker than young people to evacuate their homes. It originated from personal judgment based on their experiences.^8,14^ Wind direction has also been considered as an influential factor in understanding the risk and evacuation response of households to a Natech event.^8^ The Great East Japan Earthquake and Tsunami in 2011 revealed the lack of experience evacuating and training for the Natech events among the majority of residents.^8^

In another study has pointed out the importance of emergency evacuation in the first 72 hours after the earthquake; it can play an important role in saving the lives of survivors and reducing their damage, especially to the vulnerable group in the confronted with fire, severe aftershocks, or landslides.^25^

Thus, in the present study, it seems prevention of unnecessary evacuation can be possible via planning for the affected areas and population in determined a radius of 6.5 km area affected by the toxic gas prevention of unnecessary evacuation can be possible. However, it is necessary for preventing frequent evacuations to be considered measures, especially for the evacuated people toward the north areas direction where exposed to widespread vulnerability from the earthquake. The existence of sufficient and safe open space in the east, west, and south direction can prevent the risk of the frequent evacuation of near 61% of the population (56% of households equivalent to 56% of households). On the contrary, the insufficient capacity of northern evacuation sites can be lead to an increased risk of repeated evacuations at 38% of the population (43% of households) with a household size of 3 people (due to high structural damage). In addition, the evacuation time is limited to more than one hour for the population living in areas affected with EPRG-2 concentration,^29^ and all earthquake-safe evacuation sites are highly un-healthy and unsafe to the Natech event.

## Conclusion

Planning for the management of Natech events will be inevitable due to the probability of a major earthquake in the metropolis of Tehran, with a history of previous destructive earthquakes, high population density, adjacent to hazardous industries, and increasing of Natech events risk such as the release of toxic substances, fire, and explosion. Both short-term and long-term plans can help to cope with and resilience such events. Some short-term solutions and measures are recommended such as identifying vulnerable industries to natural hazards, identifying areas with a potential risk of Natech events, revising and updating safe routes and locations in the existing emergency evacuation maps in the ex-posed area and adjacent areas. In addition, other recommendations include informing and awareness the people living in the areas exposed to the risk of Natech events, training of appropriate individual and social behaviors when dealing with such disasters and safe and rapid evacuation procedures, development and training of emergency evacuation protocols, and organizational coordination among health personnel, response and firefighting teams, as well educational, accommodation, and underground transportation centers managers. In long term, any measures that can be lead to an increase in the possibility of evacuation and shelter-in-place are recommended such as creating incentive policies and construction regulations to help seismic retrofit of the builders and hazardous industries. Given the relative novelty of Natech risk management and Natech disaster risk reduction management, more research is needed in the emergency evacuation field. It seems to develop Research in the field of all Natech disaster management phases inevitable. The results of the present study can be useful both in understanding the Natech risks following earthquakes and prioritizing measures to addressing the challenges of emergency evacuation and the resilience increasing of surrounding communities.

**Limitation**

This study had two main limitations. At first, the research focused on one installation and one toxic gas. Despite this, in the study area, H_2_S and other toxic gas are operated in the process of several chemical installations, the research team did not have access to their information. Second, considering the disease covid-19 pandemic in the regions of Tehran, it was not possible to access pulmonary and cardiovascular disease statistics individually.

**Acknowledgements**

This research was a part of a PhD thesis approved by Shahid Beheshti University of Medical Sciences with the ethics code of IR.SBMU.PHNS.REC.1398.040 and supported by the National Iranian Oil Refining and Distribution Company. 
